# Mollaret's Meningitis: A Case Report of Recurrent Aseptic Meningitis

**DOI:** 10.7759/cureus.81065

**Published:** 2025-03-24

**Authors:** Fahim Barmak, James Issa, Danial Bajwa, Syed Hashim Ali Inam, Jason Adams, Justin Nolte, Paul Ferguson

**Affiliations:** 1 Neurology, Marshall University Joan C. Edwards School of Medicine, Huntington, USA; 2 Internal Medicine, Armed Forces Institute of Cardiology, Rawalpindi, PAK; 3 Internal Medicine, Army Medical College, Rawalpindi, PAK

**Keywords:** antiviral agents, autoimmune disorders, herpesviruses, hsv-2 meningitis, lymphocytic pleocytosis, mollaret's meningitis, recurrent aseptic meningitis

## Abstract

Benign recurrent meningitis, also known as Mollaret's meningitis, a rare form of recurrent aseptic meningitis, is often associated with herpes simplex virus type 2 (HSV-2) infection, which is characterized by fever, headache, meningeal irritation, and sterility of the cerebrospinal fluid (CSF) on examination. Although with clear diagnostic criteria, there are still many equivocal aspects of its pathogenesis and treatment. This paper presents a case report of a 40-year-old woman who experienced multiple episodes of this syndromic condition, highlighting its diagnostic challenges, clinical features, and treatment outcomes. The case reflects the importance of timely diagnosis and intervention in managing Mollaret's meningitis effectively. Furthermore, it sheds light on the complexities of its etiology and potential predisposing factors and the need for further research to better understand this condition.

## Introduction

Mollaret's meningitis is a rare, benign, recurrent form of aseptic meningitis first described by French neurologist Pierre Mollaret in 1944, who identified the condition in three patients [1,2]. This rare neurological disorder is characterized by recurring episodes of meningitis, which are generally self-limiting and benign. While the exact cause often remains unknown, the condition has been most commonly associated with infections caused by herpes simplex virus type 2 (HSV-2). Although HSV-2 is the most frequently reported cause, other infectious and non-infectious factors have also been identified as an etiologic cause of Mollaret's meningitis including Epstein-Barr virus (EBV), varicella-zoster virus (VZV), echovirus, coxsackievirus, human immunodeficiency virus (HIV), enterovirus, *Toxoplasma gondii*, as well as intracranial cysts or tumors, sarcoidosis, and systemic lupus erythematosus (SLE) [3-7]. The typical presentation includes a sudden onset of symptoms, followed by complete recovery and unpredictable recurrences [8]. Patients usually experience acute or subacute symptoms resembling classic meningitis, such as fever, severe headaches, and photophobia, but then enter symptom-free periods that may last from weeks to years. These episodes typically resolve spontaneously without the need for antibiotics or antiviral therapy. During an episode, cerebrospinal fluid (CSF) analysis often reveals lymphocyte-predominant pleocytosis, with negative cultures, polymerase chain reaction (PCR) tests, and serologies for viral causes, and normalization of lymphocytosis between attacks [9,10]. Although the literature stresses the importance of developing specific diagnostic criteria and treatment protocols for Mollaret's meningitis, there is still no consensus on its management. This report aims to highlight the clinical challenges posed by Mollaret's meningitis and provide insights into potential approaches for effective management. 

## Case presentation

A 40-year-old woman presented to the hospital in November 2024 with a two-day history of progressively worsening headache and nuchal rigidity, associated with photophobia, phonophobia, chills, body sweats, body aches, and fatigue. One month earlier, she experienced similar symptoms that resolved following treatment in the emergency department. Her clinical history revealed recurrent episodes of meningitis dating back to 2012, with subsequent presentations in 2015 and 2017. During the 2017 episode, a lumbar puncture (LP) revealed lymphocytosis, glucose of 37 mg/dL, protein of 48 mg/dL, a white blood cell (WBC) count of 29, no red blood cells (RBCs), and a positive HSV-2 PCR. These recurrent episodes raised concern for HSV-2 meningitis, given her history. Her past medical history included hypertension, attention deficit disorder (ADD), depression, uterine fibroids managed with oral contraceptive pills (OCP), gastroesophageal reflux disease (GERD), migraines, and viral meningitis. She denied smoking and reported occasional alcohol consumption. 

Initial evaluation

**Figure 1:**
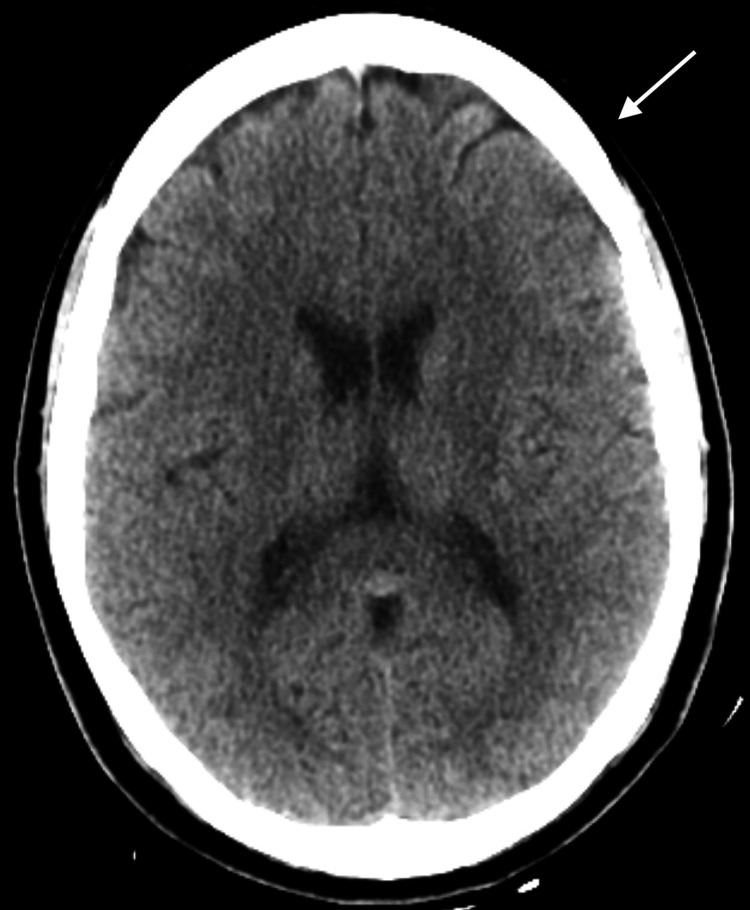
CT scan of the head (axial view) with normal ventricular size and configuration and no evidence of acute hemorrhage, ischemia, mass, mass effect, or midline displacement of structures CT: computed tomography

**Figure 2:**
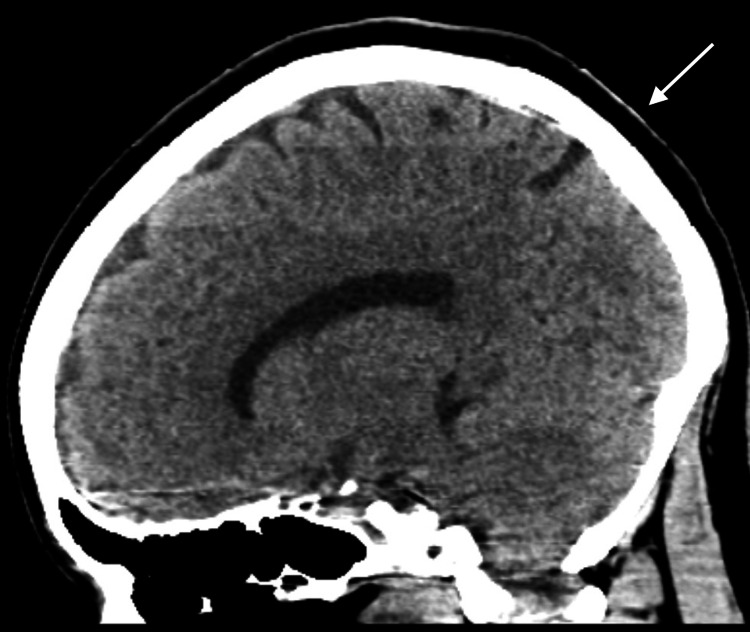
CT scan of the head (sagittal view) with no evidence of acute hemorrhage, ischemia, or mass CT: computed tomography

Upon admission, the patient underwent a thorough evaluation. Laboratory findings included a normal complete blood count (CBC) with a WBC count of 7.7×10³/µL, hemoglobin of 13.9 g/dL, and hematocrit of 42.9%. The lymphocyte count was notably elevated at 95%, suggesting a viral infection. Metabolic panel results showed a blood urea nitrogen (BUN) of 11 mg/dL, creatinine of 1.0 mg/dL, and lactic acid of 1.5 mmol/L. Hemoglobin A1c was 5.7%, indicating prediabetes. Imaging studies, including a computed tomography (CT) scan of the head and a chest X-ray, were unremarkable (Figure 1 and Figure 2). Magnetic resonance imaging (MRI) of the brain was not obtained. However, the patient had received an MRI of the brain previously at the outside facility that was reported unremarkable. She also had an MRI of the spine 10 months prior to admission that revealed L5-S1 disc herniation but nothing acute. CSF analysis revealed a colorless and clear appearance, with 1 RBC, 1 WBC, a protein concentration of 20 mg/dL, and a glucose level of 118 mg/dL. The meningitis PCR was positive for HSV-2, confirming the diagnosis of recurrent aseptic viral meningitis. 

Emergency department visit (one month prior)

Approximately one month prior to this admission, the patient had presented to the emergency department with similar symptoms, including headache and nuchal rigidity. She was treated with diphenhydramine (PO 25 mg), ketorolac (IM 15 mg), dexamethasone (IM 8 mg), and normal saline (IV 500 ml) resulting in symptom resolution. She was discharged after a short stay and was stable at the time of discharge.

Hospital course

The patient was started on intravenous medications for two days, including dexamethasone (IV 10 mg every six hours), ceftriaxone IV 2 g every 12 hours), vancomycin (IV 1250 mg every eight hours), and acyclovir (IV 595 mg every eight hours), to cover potential viral and bacterial causes of meningitis. Diabetes management was initiated after a blood glucose level in the 400s was noted, and 10 units of Lantus insulin were administered. The patient was subsequently discharged on metformin (500 mg daily) for her prediabetes. Oral antiviral therapy with valacyclovir (PO 1 g every eight hours daily for 10 days) was initiated to target the HSV-2 infection. Antibacterial therapy was discontinued after negative blood cultures and meningitis PCR results. Infectious Disease (ID) specialists were consulted, and further testing including CSF gram stain and culture were negative, and blood cultures remained negative. 

Subsequent admission

Forty days after the initial presentation, the patient returned to the hospital with persistent headaches and nuchal rigidity lasting for four days. She described the headache as similar to previous episodes associated with herpes meningitis. Laboratory findings at this visit revealed a WBC of 12×10³/µL, hemoglobin of 13.0 g/dL, platelet count of 457×10³/µL, and lactic acid of 1.6 mmol/L, and blood culture did not reveal any pathogen. However, CSF analysis was not performed at this visit. The recurrent nature of similar presentations further supported the diagnosis of recurrent aseptic viral meningitis.

Outcome and follow-up

The patient's symptoms improved with treatment, and she was discharged in stable condition. She was given recommendations for follow-up care to monitor her glucose levels and manage her underlying conditions. A follow-up with neurology was scheduled to monitor for potential recurrence of aseptic meningitis and ensure the optimal management of her chronic conditions. During the follow-up visit at the neurology clinic and primary care physician clinic, the patient reported no further recurrence of similar symptoms.

## Discussion

Mollaret's meningitis was first described by French neurologist Pierre Mollaret in 1944 as a form of recurrent aseptic meningitis, the cause of which was initially unknown [11]. This rare syndrome is characterized by repeated episodes of fever and meningeal irritation, which typically resolve within 2-5 days. Unlike other forms of viral meningitis, Mollaret's meningitis stands out due to its recurrent nature, with patients experiencing up to 15 recurrences. These episodes are often separated by symptom-free intervals that can last from weeks to months [12]. Our patient has experienced multiple and recurrent hospital admissions with similar presentations. 

The recurrent nature of the condition, along with its symptom overlap with other types of meningitis, presents unique diagnostic challenges. Mollaret's meningitis may occur over the course of months or even years, with long asymptomatic periods in between episodes. Various conditions have been identified as potential triggers for these recurrences, including para-meningeal infections such as sinusitis and mastoiditis, post-traumatic bacterial meningitis, and certain medications [11]. 

The pathogenesis of Mollaret's meningitis remains incompletely understood, often considered a disease of unknown etiology. However, research has identified several potential contributors, including viral infections, autoimmune disorders, genetic factors, and environmental triggers. Herpesviruses, particularly HSV and EBV, are frequently implicated in the condition, with HSV-2 being the most common causative agent [7,12]. These viruses are capable of establishing latent infections in the central nervous system (CNS), with recurrent meningitis occurring due to retrograde seeding of the virus from sensory neurons in the dorsal root ganglia. 

HSV-2, in particular, is known to remain dormant in the sensory neurons following the initial infection. Under certain conditions, such as immunosuppressive states, physical or emotional stress, or fever, the virus can reactivate, leading to recurrent episodes of meningitis [11]. This ability to reactivate in the CNS is a hallmark of herpesviruses, making them prime candidates for recurrent meningeal involvement [12]. Additionally, other herpesviruses such as HSV-1, VZV, and EBV have also been implicated, although less commonly. 

In addition to viral causes, Mollaret's meningitis has been associated with non-infectious etiologies. Chemical meningitis, linked to conditions such as dermoid cysts, and certain benign CNS tumors, including craniopharyngiomas and epidermoid cysts, have also been recognized as potential contributors to the condition [13]. These non-infectious causes may trigger an inflammatory response in the meninges, mimicking the recurrent nature of the disease. 

Recent studies have also highlighted the role of autoimmune disorders in the development of recurrent meningitis. Diseases such as Behçet's disease, sarcoidosis, and SLE, particularly with the presence of antiphospholipid antibodies, have been associated with episodes of Mollaret's meningitis [7]. These autoimmune conditions may predispose individuals to recurrent CNS inflammation and meningitis, likely through abnormal immune responses. 

Furthermore, genetic factors play a significant role in the pathogenesis of Mollaret's meningitis. A genetic predisposition to the disease has been suggested, with associations to familial Mediterranean fever (FMF) and mutations in specific genes, such as TLR3 and UNC-93B, which are involved in immune regulation [7]. Mutations in these genes may impair the body's ability to regulate the inflammatory response, leading to an exaggerated immune reaction in the brain and spinal cord, which could contribute to the recurrent nature of the disease. Additionally, mutations in the TNFAIP3 gene, which regulates inflammatory processes, have also been linked to an increased susceptibility to recurrent meningitis. In our patient, there was no history of autoimmune disorders, and genetic testing was not pursued, but further studies are warranted to explore genetic predispositions to the disease. Environmental factors may also play a role in disease recurrence. 

Together, these findings suggest that Mollaret's meningitis may arise from a complex interplay of viral infections, autoimmune disorders, genetic predispositions, and possibly environmental factors. Despite these advances, further research is needed to fully understand the underlying mechanisms and to refine diagnostic and treatment strategies for this enigmatic condition. 

Diagnosing Mollaret's meningitis can be challenging due to the lack of specific diagnostic tests. As such, diagnosis typically relies on a combination of clinical signs, laboratory tests, and neuroimaging studies. Traditional diagnostic criteria focus on the absence of identifiable etiological agents, which can make it difficult to pinpoint the exact cause of recurrent episodes. However, the detection of HSV DNA in the CSF during recurrent episodes can strongly support the diagnosis. HSV DNA is present in up to 82% of CSF samples collected 2-5 days after symptom onset [7]. 

It is important to note that mucocutaneous lesions are not required for diagnosis, as up to 50% of patients may not report prior genital herpes lesions [14]. CSF analysis plays a crucial role in diagnosis, typically showing a predominance of mononuclear cells, including large monocytes with convoluted nuclei, known as Mollaret cells. These cells are a hallmark of the disease and can be observed early in the course of the illness [3,12]. In our case, CSF analysis showed positive HSV-2 PCR. 

In addition to CSF analysis, neuroimaging studies may provide further insight, often revealing inflammation in the brain or spinal cord, which can contribute to recurrent episodes of meningitis [15]. 

In a case report involving four patients, the authors highlighted an association between herpesvirus infections and Mollaret's meningitis, with HSV or viral DNA detected in the CSF through PCR amplification. Three of the four patients tested positive for HSV-2 DNA during meningitis episodes, while cultures from CSF and serum were negative for bacteria, mycobacteria, viruses, and fungi [12]. Supporting these findings, a retrospective case series by Kupila et al. and Tedder et al. found HSV DNA in 79-85% of CSF samples, predominantly from HSV-2. They noted that negative HSV DNA results could occur due to rapid viral clearance from the CSF or delays in patient presentation and sample collection [16,17]. 

HSV-2 is particularly notable in the context of Mollaret's meningitis, with the condition reported as a complication of primary genital herpes in 36% of women and 11% of men. Recurrent HSV-2 meningitis can occur in 19-27% of cases, particularly in individuals who are seronegative for HSV-1 [7]. In addition to HSV-2, other herpesviruses have also been implicated in Mollaret's meningitis. For example, a case report from Japan identified VZV as a potential cause, based on genetic analysis, anti-VZV IgG in the CSF, a VZV IgG index, and elevated interleukin-1 beta (IL-1β) levels, despite undetectable VZV DNA in the CSF [18]. 

Furthermore, in a study by Prandota, latent cerebral toxoplasmosis was described as another potential trigger for Mollaret's meningitis. Prandota noted that factors such as cytokines, nitric oxide, and reactive oxygen species could influence the severity of the disease in such cases [19]. 

The diagnosis of Mollaret's meningitis is further guided by Bruyn et al.'s criteria (1962) (Table 1) and Galdi's modified criteria (1979) (Table 2), which reflect the disease's complex and variable clinical presentation [20,21].  

**Table 1:** Bruyn et al.'s criteria for the diagnosis of Mollaret's meningitis CSF: cerebrospinal fluid Table Credits: Author's creation based on Bruyn et al. [20]

Bruyn et al.'s criteria for the diagnosis of Mollaret's meningitis	
1. Recurrent episodes	The patient experiences recurrent episodes of fever and symptoms indicative of meningismus.
2. CSF pleocytosis	Each episode is characterized by pleocytosis in the CSF, indicating an inflammatory response.
3. Symptom-free periods	There are prolonged symptom-free intervals between episodes, lasting weeks or months.
4. No lingering symptoms	After each episode, patients recover fully without neurological sequelae.
5. Absence of causative microbes	No identifiable causative agent is found during diagnostic evaluation.

**Table 2:** Galdi's modified criteria for the diagnosis of Mollaret's meningitis CSF: cerebrospinal fluid Table Credits: Author's creation based on Galdi [21]

Galdi's modified criteria for the diagnosis of Mollaret's meningitis	
1. Fever may not be present	Unlike Bruyn et al.'s criteria, Galdi acknowledged that fever might not always be present during episodes.
2. Neurological abnormalities	Transient neurological abnormalities, apart from meningismus, occur in about 50% of cases.
3. Variable symptom-free periods	The duration of symptom-free intervals between attacks can vary significantly, from days to years.
4. CSF gamma globulin fraction	Increased gamma globulin levels may be observed in the CSF during episodes, an additional feature not included in Bruyn et al.'s criteria.

Table 3 compares Mollaret's meningitis and the etiologic causes of recurrent aseptic meningitis.

**Table 3:** Comparison of Mollaret's meningitis and the etiologic causes of recurrent aseptic meningitis HSV: herpes simplex virus; SLE: systemic lupus erythematosus; EBV: Epstein-Barr virus; VZV: varicella-zoster virus; HIV: human immunodeficiency virus; AIDS: acquired immunodeficiency syndrome; CD4: cluster of differentiation 4 (a glycoprotein found on the surface of immune cells, crucial for immune response); RNA: ribonucleic acid; DNA: deoxyribonucleic acid; PCR: polymerase chain reaction; anti-dsDNA: antibody to double-stranded deoxyribonucleic acid (a marker often associated with autoimmune diseases like systemic lupus erythematosus); ACE: angiotensin-converting enzyme (often elevated in conditions like sarcoidosis); HLA-B51: human leukocyte antigen B51 (a genetic marker associated with Behçet's disease); WBC: white blood cell; CSF: cerebrospinal fluid; MRI: magnetic resonance imaging; CNS: central nervous system Table Credits: Author's creation

Characteristic	HSV	Behçet's disease	Sarcoidosis	SLE	Chemical meningitis	EBV	VZV	Echovirus	Coxsackievirus	HIV	Enterovirus	Toxoplasma gondii	Intracranial cysts/tumors
Etiology	HSV (HSV-2, the most common cause) infection	Autoimmune, associated with systemic inflammation	Chronic inflammatory disorder, granulomas	Autoimmune, associated with systemic inflammation	Reaction to irritants (e.g., drugs, contrast agents)	Herpesvirus (EBV)	Herpesvirus (VZV)	*Enterovirus* family (e.g., *Echovirus*)	*Enterovirus *family (e.g., *Coxsackie*)	HIV infection	Enterovirus (e.g., EV-D68)	*Toxoplasma gondii *(protozoan)	Intracranial cysts, tumors, or masses
Clinical presentation	Recurrent episodes of fever, headache, neck stiffness, nausea, vomiting	Recurrent oral/genital ulcers, eye involvement, skin lesions	Non-specific symptoms, can present with headaches and cranial nerve palsies	Malar rash, joint pain, other systemic symptoms	Symptoms after exposure to irritants or chemicals	Mononucleosis-like symptoms, fever, sore throat	Dermatomal rash, acute meningitis, encephalitis	Acute aseptic meningitis, often in infants and children	Meningitis with other viral illnesses, including hand-foot-mouth disease	Acute retroviral syndrome, chronic HIV-related complications	Aseptic meningitis, acute febrile illness	Meningitis with systemic infection, often in immunocompromised hosts	Can present with headache and focal neurological symptoms depending on the location of the cyst/tumor
CSF findings	Elevated WBC count (pleocytosis), normal glucose, normal protein	Mild pleocytosis, normal glucose, normal protein	Pleocytosis with possible granulomas, elevated ACE levels	Elevated WBC count, possible cytology for tumor or cyst	Pleocytosis, but normal glucose and protein	Lymphocytic pleocytosis, normal glucose, normal protein	Pleocytosis with lymphocytic predominance, normal glucose, normal protein	Pleocytosis, normal glucose, normal protein	Pleocytosis, normal glucose, normal protein	Pleocytosis, low CD4 count in the CSF in advanced HIV	Pleocytosis, normal glucose, normal protein	Elevated WBC count, normal glucose, normal protein	Variable: pleocytosis, elevated WBC, normal glucose and protein
MRI findings	Normal or non-specific changes during episodes	May show CNS involvement (brainstem, spinal cord)	Granulomas in the brain or spinal cord, meningeal thickening	May show signs of vasculitis, CNS involvement (e.g., stroke-like lesions, cerebral edema)	Non-specific findings, may show contrast enhancement due to chemical irritants	May show evidence of encephalitis, lymphocytic meningitis	Can show herpesvirus encephalitis with brainstem or temporal lobe involvement	Non-specific, occasionally brain involvement	Non-specific, may show involvement of the spinal cord or brainstem	CNS changes in advanced HIV (e.g., HIV-associated dementia)	Non-specific, may show meningeal enhancement	May show space-occupying lesions (e.g., ring-enhancing lesions in toxoplasmosis)	Mass effect, space-occupying lesions (e.g., cysts, tumors)
Cerebral imaging	Often unremarkable between episodes	May show brainstem or spinal cord lesions	Granulomas in the affected brain or spinal cord	May show cerebral or spinal cord vasculitis, infarcts	No specific imaging changes	Can show encephalitis or lymphocytic meningitis	MRI may show lesions related to VZV infection	Mild to moderate changes in neonates/infants	Mild to moderate changes, may show spinal cord lesions	Normal early on, with progression in advanced stages	Often shows mild to moderate brain or meninges involvement	Can show ring-enhancing lesions, hydrocephalus in immunocompromised	May show space-occupying lesions (e.g., cysts or tumors)
Differentiation from other conditions	History of recurrent, self-limited episodes, HSV PCR in the CSF (if available)	Clinical features of ulcers, eye involvement, positive HLA-B51, systemic involvement	Granulomas, elevated ACE levels, biopsy may be needed	ANA, anti-dsDNA, clinical signs of SLE	History of chemical exposure, CSF analysis showing no infectious agent	Positive monospot or EBV PCR, heterophile antibodies	PCR for VZV, clinical features of herpes zoster	PCR for enterovirus, often seen in children with viral prodrome	PCR for coxsackievirus, clinical correlation with hand-foot-mouth disease	HIV RNA levels in the CSF, CD4 count	PCR for enterovirus, PCR for specific serotypes	Toxoplasma serology, PCR, biopsy of the affected tissue	Imaging, biopsy of cyst or tumor, histology
Treatment	Supportive care, potential antiviral therapy (acyclovir) during active episodes	Immunosuppressive therapy (e.g., corticosteroids, biologics)	Corticosteroids, immunosuppressive therapy	Immunosuppressive therapy for SLE, corticosteroids	Removal of irritants, steroids, or anti-inflammatory agents	Supportive care, antivirals (e.g., acyclovir) if encephalitis	Antiviral therapy (e.g., acyclovir), corticosteroids for severe cases	Supportive care, usually resolves in 7-10 days	Supportive care, antivirals if needed (e.g., for severe disease)	Antiretroviral therapy, opportunistic infection management	Supportive care, antivirals (e.g., acyclovir for encephalitis)	Antitoxoplasmic therapy (e.g., pyrimethamine, sulfadiazine)	Surgery, drainage if needed, chemotherapy for tumors
Prognosis	Generally favorable with treatment; relapses can occur	Chronic disease with variable prognosis; can be severe if untreated	Chronic, but can enter remission	Variable; can be severe or result in long-term neurological complications if untreated	Often resolves once chemical irritants are removed	Most patients recover fully; encephalitis can lead to sequelae	Most patients recover fully; severe cases may require antivirals	Generally self-limiting; rarely severe	Generally self-limiting; complications rare	Chronic condition; can lead to AIDS without treatment	Usually self-limiting; rare complications like encephalitis	Typically good prognosis if treated, but can cause severe complications in immunocompromised	Depends on the size, location, and type of cyst/tumor

The optimal treatment for Mollaret's meningitis remains unclear, largely due to the uncertain etiology of the disease. Some reports suggest improvement with chronic valacyclovir treatment, while others question the role of antiviral drugs in managing the condition [6]. Long-term suppressive antiviral therapy with acyclovir or valacyclovir has shown promise in reducing the frequency and severity of HSV-2-associated Mollaret's meningitis, but high-quality evidence supporting this approach is lacking. Both acyclovir and valacyclovir are generally well tolerated and can help reduce the frequency of attacks. However, other medications, such as colchicine and indomethacin, have been tried, though their effectiveness remains unproven [7,15]. 

A randomized controlled trial (RCT) involving 101 patients with acute or recurrent HSV-2 meningitis compared valacyclovir treatment to a placebo. Initially, no significant difference was found between the two groups. However, after one year, the recurrence rate of HSV-2 meningitis was significantly higher in the valacyclovir group after treatment was discontinued, with a hazard ratio of 3.29 (95% CI: 10.06-10.21) [22]. Some studies suggest that antiviral therapy may benefit immunocompromised patients but provide limited benefit in immunocompetent individuals [23]. 

In a case report from Japan, indomethacin was suggested as a potential treatment for acute episodes and prevention of recurrence in Mollaret's meningitis. The administration of indomethacin resulted in reduced levels of IL-6 and TNF-α in both CSF and serum. However, the author could not definitively attribute symptom relief to the drug, as the disease typically resolves within 3-5 days, regardless of treatment [24]. 

For HSV-2 meningitis, the Centers for Disease Control and Prevention (CDC) recommends intravenous acyclovir (5-10 mg/kg every eight hours) until improvement is seen, followed by a switch to oral valacyclovir (1000 mg three times daily) for 10-14 days. The CDC also suggests oral valacyclovir alone from presentation to completion of treatment in patients with prior documented meningitis recurrence due to HSV-2, but does not recommend suppressive therapy with valacyclovir. Despite the self-limiting nature of the disease and the usual clinical course of spontaneous resolution, withholding intravenous acyclovir and opting only for supportive treatment may not be appropriate in certain clinical circumstances [14]. Our patient received valacyclovir (PO 1 g every eight hours daily for 10 days) to address the HSV-2 infection. Symptomatic treatment with supportive care was also employed. 

While data on the prognosis of Mollaret's meningitis is scarce, the disease generally resolves within 3-5 days, with most patients recovering between episodes. In many cases, the disease disappears entirely after 3-5 years, although reports of longer durations and more frequent episodes exist [25,7]. Long-term complications are rare, but some patients may experience post-meningitis migraines [6]. 

## Conclusions

Mollaret's meningitis remains a challenging diagnosis due to its recurrent and episodic nature. The association with HSV-2 reactivation is well established, and viral testing during episodes of meningitis is essential for confirmation. While the treatment of acute episodes focuses on supportive care and antivirals, further research is needed to better understand the pathogenesis of the disease and determine optimal long-term management strategies. Clinicians should maintain a high index of suspicion in cases of recurrent aseptic meningitis, particularly in patients with a history of HSV-2 infections. 
